# Lead/Drug Discovery from Natural Resources

**DOI:** 10.3390/molecules27238280

**Published:** 2022-11-28

**Authors:** Zhihong Xu, Barrett Eichler, Eytan A. Klausner, Jetty Duffy-Matzner, Weifan Zheng

**Affiliations:** 1 Department of Chemistry and Biochemistry, Augustana University, 2001 S Summit Ave., Sioux Falls, SD 57197, USA; 2 Institute of Interventional & Vascular Surgery, Tongji University, Shanghai 200072, China; 3 Department of Pharmaceutical Sciences, South College School of Pharmacy, 400 Goody’s Lane, Knoxville, TN 37922, USA; 4 Biomanufacturing Research Institute and Technology Enterprise, North Carolina Central University, 1801 Fayetteville St., Durham, NC 27707, USA; 5 Division of Chemical Biology and Medicinal Chemistry, Eshelman School of Pharmacy, University of North Carolina at Chapel Hill, Chapel Hill, NC 27599, USA

**Keywords:** antibacterial, anticancer, antimalarial, antiviral, computer-assisted, drug discovery, lead discovery, natural product

## Abstract

Natural products and their derivatives have been shown to be effective drug candidates against various diseases for many years. Over a long period of time, nature has produced an abundant and prosperous source pool for novel therapeutic agents with distinctive structures. Major natural-product-based drugs approved for clinical use include anti-infectives and anticancer agents. This paper will review some natural-product-related potent anticancer, anti-HIV, antibacterial and antimalarial drugs or lead compounds mainly discovered from 2016 to 2022. Structurally typical marine bioactive products are also included. Molecular modeling, machine learning, bioinformatics and other computer-assisted techniques that are very important in narrowing down bioactive core structural scaffolds and helping to design new structures to fight against key disease-associated molecular targets based on available natural products are considered and briefly reviewed.

## 1. Introduction

Traditional medicine has a long history of clinical practice in many countries due to the fact that nature provides a rich source of active ingredients for multi-target therapies. Natural products are also more affordable to most patients, along with the convenience of self-medication. However, concerns have been associated with insufficient study of herbal/animal (and other crude) products’ clinical efficacy and safety, and their purity and potency are not regulated [1]. Some components in natural product mixtures may cause severe adverse effects and drug–drug interactions [1,2]. For example, some herbal supplements have caused hepatotoxicity mediated by the modulation of cytochrome P450 [3]; the neurotoxic *Atropa belladonna* may cause death in children [4]; some constituents can cross the placenta and reach the fetus to cause embryotoxicity, teratogenic and abortifacient effects [5], and some compounds in medicinal plants will generate subacute and chronic toxicities with carcinogenic and endocrine-disrupting effects [6]. Kharchoufa et al. reported that toxic compounds in 287 medicinal plants used in the northeastern region of Morocco mainly include alkaloids, glucosides, terpenoids, protides, and phenolics [7].

The United States (U.S.) Food and Drug Administration (FDA) Center for Drug Evaluation and Research (CDER)’s approval of crude natural drugs (extracts) is very restricted, and processing takes longer. Among a number of Traditional Chinese Medicine (TCM) companies filing investigational new drug (IND) applications, only two botanicals have been approved as drugs [8,9,10] for clinical care: Veregen, a green tea extract, for the treatment of external genital and perianal warts in 2006, and Mytesi (originally Fulyzaq), an anti-diarrheal (non-infectious) medication for human immunodeficiency virus (HIV)-infected adults in 2012. Eight more TCMs are approved by the U.S. FDA for Phase II/III clinical trials currently, but with very few promising results for future drug development [8]. The complexity of the composition and the uncertainty of the active ingredients and mechanisms of a number of crude TCM mixtures make the evaluation of their efficacy, safety and quality control very challenging under current clinical evaluation systems. In addition, the dosing formulation and routes of TCM have prolonged the investigation time needed for drug approval, as TCM has been mostly administrated orally and special attention has been paid to the combination and processing of TCM [8] since ancient times. To avoid unpredictable adverse effects/toxicities and for the effectiveness of drug candidate improvement, scientists all over the world have devoted intensive efforts to isolating (bioassay-directed) and identifying bioactive pure compounds from nature. This is often time-consuming, but, with the help of numerous modern techniques, the discovery of bioactive compounds and the later stages of druggable lead selection and mechanistic studies can be accelerated. Lead compounds have pharmacological or biological activities likely to be therapeutically useful and they are leading candidates for new drugs. The studies of the mechanisms of action and toxicity of pure leads isolated from natural resources at molecular levels are very important, which will give the direction for lead improvement and further drug development. Several well-known drugs originally discovered from TCM include artemisinin, huperzine A and berberine. 

**Figure 1:**
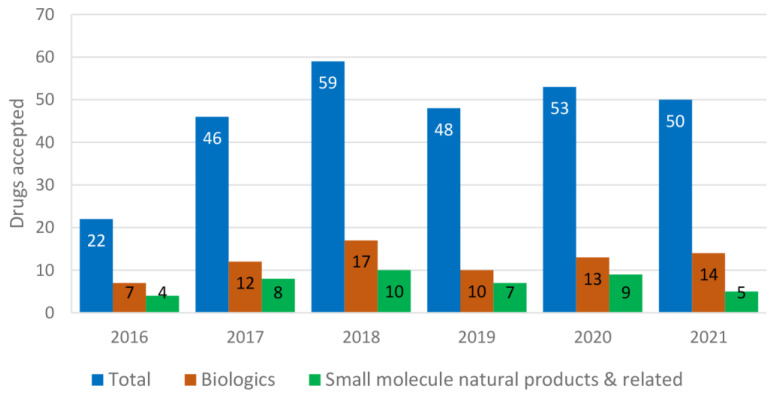
Drugs recently approved by the U.S. FDA (2016–2021).

Out of the total 209 drugs approved by the U.S. FDA from 2000 to 2008, 9.09% were used for the treatment of cardiovascular disorders and 12.91% for neurological disorders. Antibiotics (5.26%) and antivirals (5.74%) were least contributed, whereas anticancer drugs (11.96%) and biologics (7.17%) remained constant [11]. Biologics include antibodies, antibody–drug conjugates, enzymes, pegylated proteins, etc. Of 302 drugs approved from 2009 to 2017, 5.29% were used for cardiovascular disorders, 9.93% for neurological disorders, 5.29% for antibiotics, 5.96% for antivirals, 17.54% for anticancer agents and 15.56% for biologics [11]. Among these approved drugs, most were natural products or related [12]. It is obvious that the use of fast-tracked, accelerated approval and priority review programs has been steadily increasing since 2000 [11]. The comparison of each year’s (2016–2021) total drugs approved vs. the number of biologics and the number of natural-product-related small molecules is shown in Figure 1. Biologics (seven monoclonal antibodies) accounted for 32% drugs approved for 2016 (a total of 22), and four small-molecule natural-product-related agents (amino acid derivatives briviac, anumni, and netspot, and a semisynthetic bile acid ocaliva) were approved [13]. Among the 46 drugs approved in 2017, 12 were biologics and 8 were amino acids (betrixaban, safinamide, netarsudil, valbenazine, deutetrabenazine) and other natural-product-based drugs (ertugliflozin, deflazacord, naldemedine) [14]. For 2018, out of the 59 drugs approved, 17 were biologics and 10 were natural-product-based (eravacycline, omadacycline, sarecycline, migalastat, plazomicin, moxidectin, rifamycin SV, the steroid family ethinylestradiol and segesterone, cannabidiol, fatty acid emulsion Omegaven™) [15]. Among the 48 new drugs approved for 2019, 10 were biologics and 7 were inspired by natural products (cefiderocol, istradefylline, fedratinib, allopregnanolone or brexanolone, fluorodopa, solriamfetol, lefamulin) [16]. The 53 approvals in 2020 were divided between 13 biologics and 40 new chemical entities, including nine natural-product-based small molecules (artesunate, lurbinectedin, lactitol, fluoroestradiol F18, clascoterone, triheptanoin, decitabine and cedazuridine, remdesivir) [17]. Among the 50 drugs approved in 2021, 14 were biologics, and only five were natural-product-based small compounds (drospirenone and estetrol, samidorphan, maribavir, ibrexafungerp) [18]. Up to October 2022, there were 28 new drug approvals [19], including nine biologics and less than five natural-product-based small molecules.

Although there have been a number of drugs brought to market for clinical use every year, drug resistance, adverse effects, and potency are still the major concerns. Drug discovery is at an all-time-high priority globally, and the success or failure of these innovations can have a significant impact on human health. Preclinical stages of drug discovery include project selection and target validation, virtual high-throughput screening, and lead discovery and optimization before a drug candidate can be developed for clinical trials. Potency and safety are the major concerns for a successful drug candidate. Nature is a rich resource for research project selection, and it has been proven to be the most productive source of lead discovery [20]. Bioactive herbs and other natural-product-related projects are mainly chosen based on traditional medical experience and/or high-throughput cell-based screening. Natural-product-based new molecular target discovery [21] and the development of high-throughput screening to identify small-molecule inhibitors of molecular targets (e.g., [22]) have sped up the research in natural product chemistry. Nature-based drugs include agents isolated from plants (aspirin, morphine, artemisinin, vincristine, paclitaxel, camptothecin, elliptinium, galantamine, huperzine, β-elemene [23], etc.), microbes (penicillins, cephalosporins, erythromycin, streptomycin, daptomycin, tetracycline, neomycin, chloramphenicol, etc.), and animals or animal toxins [24] (enoxaparin, exenatide, ziconotide, etc.), as well as semi-synthetic/synthetic identities inspired by natural origin compounds (e.g., lisinopril, captopril, everolimus, azithromycin, tigecycline, micafungin, caspofungin, artemether, stavudine, irinotecan, valrubicin, cabazitaxel, vinorelbine, etc.). The therapeutic uses of these diversified chemical structures include anticancer, antiviral, antibacterial, antifungal, antiparasitic, anti-diabetic, immunomodulating, etc., among which around two thirds of the antibiotics currently used in clinics are from fungi and actinobacteria. 

Common natural products isolated include terpenoids, alkaloids, flavonoids, lignans, steroids, saponins, peptides/proteins, nucleosides/nucleotides, polysaccharides, phenolic and other miscellaneous compounds. More than 40 cyclic peptide drugs have been clinically approved over the past few decades, and the majority were derived from natural products [25]. Alkaloids account for ~20% of the reported secondary metabolites from plants, which play an essential role in natural defense. Therapeutically, alkaloids are well known to be anesthetics, cardioprotective and anti-inflammatory agents [26], and potent anticancer and antiviral agents ([27,28], Section 2.1 and Section 2.2). One typical example is quinoline and its derivatives, which present multiple bioactivities, such as antibacterial, antifungal, antimycobacterial, antiviral, anti-protozoal, antimalarial, anticancer, cardiovascular/central nervous system (CNS) effects, antioxidant, anticonvulsant, analgesic, anti-inflammatory, anthelmintic, and miscellaneous activities [29]. Currently, at least 60 plant-derived alkaloids have been approved as drugs for clinical use. The mechanisms of some alkaloids have been very well studied. For example, cephalomannine was found to exert inhibitory effects in hypoxic lung cancer cells via the inhibition of the interaction between apurinic/apyrimidinic endonuclease-1 (APEX1) and hypoxic-induced factor-1α (HIF-1α) [30]. Another typical example is sanguinarine, a plant-derived cancer prevention and treatment alkaloid; it impedes tumor metastasis and development by disrupting a wide range of cell signaling pathways, and molecular targets such as B-cell lymphoma-2 (BCL-2), mitogen-activated protein kinases (MAPKs), protein kinase B (Akt protein), nuclear factor kappa B (NF-κB), reactive oxygen species (ROS), and microRNAs (miRNAs) [31].

As most natural-product-based drugs currently undergoing clinical trials or preclinical development [32] are derived mainly from lead compounds isolated from plants and microbials, among which most candidates are anticancer agents and anti-infectives, we would like to focus this review on recent natural product research in the areas of anticancer, antiviral, antibacterial and antimalarial lead/drug discovery. Anticancer and antiviral agents reported in this paper are selected in consideration of their potency, novel structures, and/or new activities and/or new mechanisms, with a focus on overcoming drug resistance and adverse effects; meanwhile, antibacterial agents are considered for their unique structures and/or chemical composition, and their clinical importance. Here, antimalarial natural drug research will be used as an example to address recent natural-product-based studies in the anti-parasitic therapeutic area, where fewer drugs have been discovered over time. *Plasmodium falciparum* is of special concern as it is the deadliest and causes the majority of malarial cases. K1 strands of this organism are of interest since they have developed multidrug resistance (MDR). The potent novel compounds are of particular interest because they do not share structural similarities with known antimalarial drugs and are thus of interest in developing new antimalarial treatments. 

**Figure 2:**
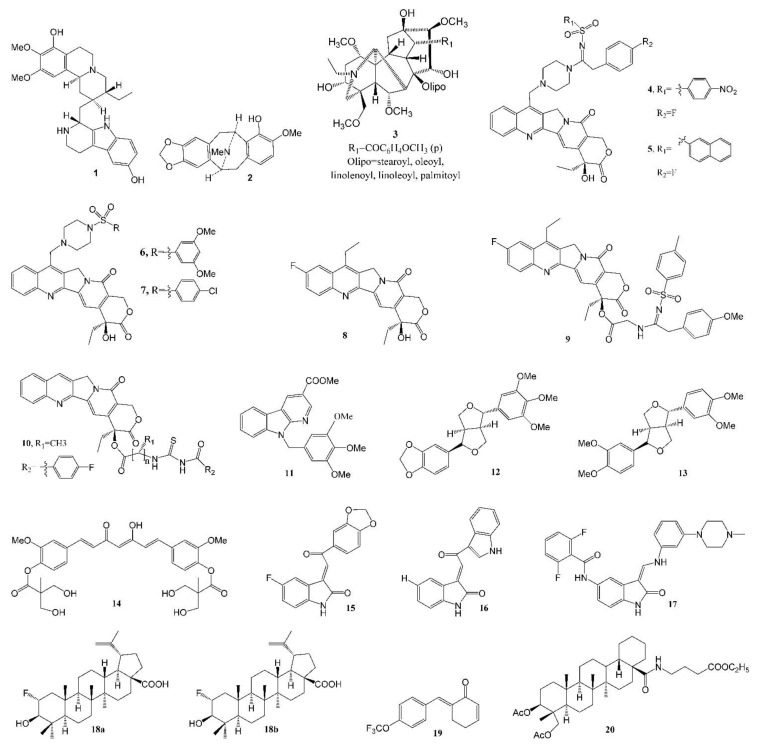
Representative antitumor compounds reported by the Lee laboratory (2016–2021).

**Figure 3:**
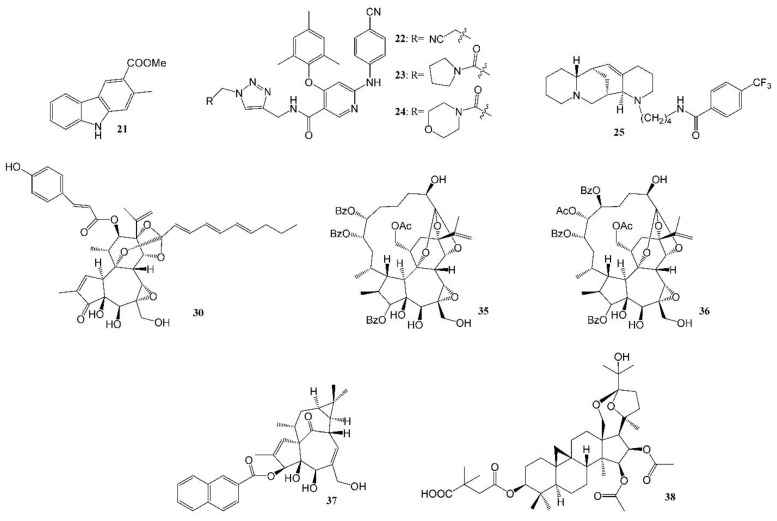
Representative anti-HIV compounds reported by the Lee laboratory (2016–2021).

**Figure 4:**
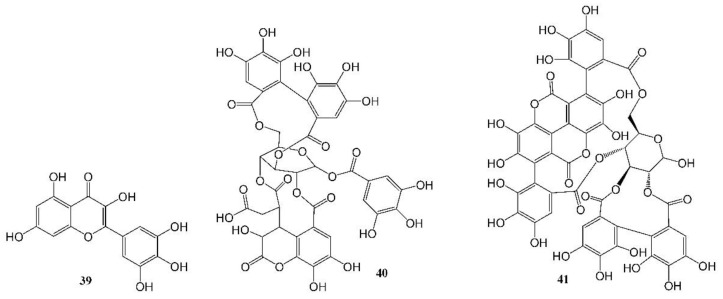
Anti-SARS-CoV-2 (COVID-19) natural products.

**Figure 5:**
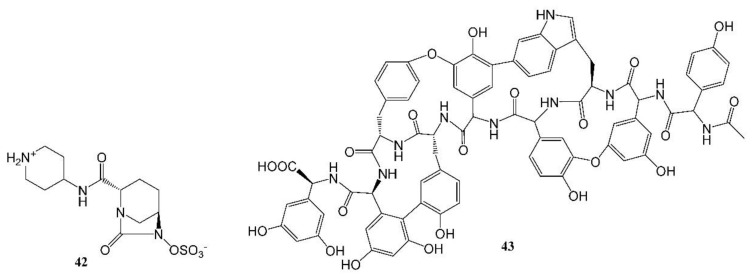
Structures of recently approved antibacterial drugs.

**Figure 6:**
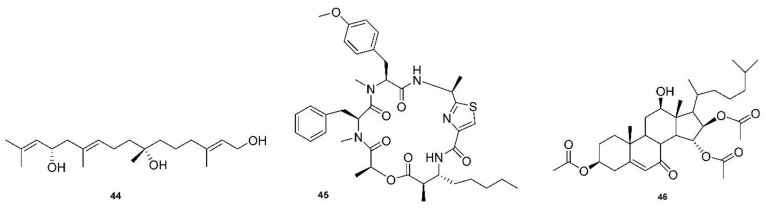
Novel reported antimalarial natural products from marine sources.

**Figure 7:**
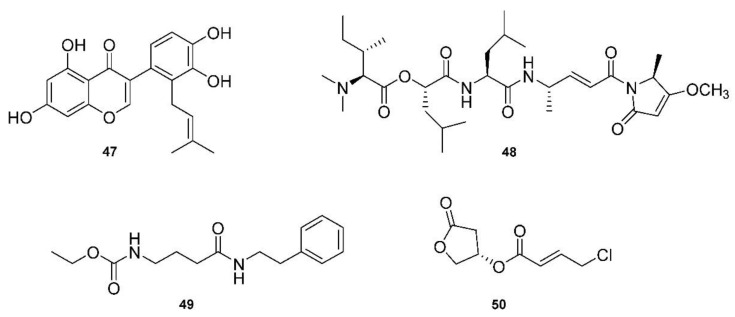
Structurally important bioactive natural products from marine sources.

The ocean is a rich and important source for a number of structurally unusual bioactive compounds, so this report will also briefly summarize recent lead discovery in marine chemistry based on potency. Computer-assisted drug discovery from natural resources is briefly summarized here as well. Most representative bioactive agents reported in this paper are listed in Table 1, Tables 2–3, and structures are shown in Figures 2–7. 

## 2. Selected Bioactive Natural Products and Analogs

### 2.1. Antitumor Drugs/Agents

Anticancer drug discovery and development are very important to human health, as cancer is one of the major diseases that causes mortality globally. Natural product structures have been shown to be very influential in the anticancer drug discovery field. The early success of finding cancer drugs of natural origin has prompted significant natural product research in the anticancer area. For example, from 1946 to 1980, of the 75 small molecules, 40% were isolated natural products and 53.3% were natural-product-derived compounds, and a total of ~65% of these natural products and related semi-synthetic molecules as drugs are still in use [53]. Anticancer drugs are mainly derived from microbe and terrestrial plant products [54,55]. Certain microbials, such as *Streptomycetes* from mangrove environments, have the ability to synthesize diverse anticancer compounds [56]. Terrestrial plants are a rich source of major nature-derived drugs used for chemotherapy. All cancer chemotherapy drugs have problems of high toxicity or adverse effects, as well as the development of drug resistance over time, and combination therapy is a partial solution to address these concerns. The diverse structures from nature may provide promising leads to overcome these problems.

The Lee group at the Eshelman School of Pharmacy of University of North Carolina at Chapel Hill (UNC-CH) discovered a number of potent anticancer natural products over the past five decades. Prof. Kuo-Hsiung Lee published approximately 950 articles and around 120 patents in the area of natural product chemistry during his 50 years of service at UNC-CH, mainly focused on the discovery of potent new anticancer and anti-HIV agents from isolated natural products and derived analogs. Table 2 lists the representative anticancer agent bioassay data from the Lee laboratory in the last six years (2016–2021), and compound structures are shown in Figure 2. 

An apoptosis-inducing tetrahydroisoquinoline alkaloid, 8-hydroxytubulosine **1** (Figure 2, Table 1 and Table 2), was isolated from the leaves of the endangered tropical plant *Alangium longiflorum*. This alkaloid displayed broad-spectrum inhibition at submicromolar levels against most tested tumor cell lines, except for drug-transporter-overexpressing cells [33]. *Bousigonia mekongensis* contains various monoterpenoid indole alkaloids. Several known aspidosperma-type monoterpenoid indole alkaloids [34], 3α-acetonyl-tabersonine, 14,15-α-epoxy-11-methoxytabersonine, lochnerinine and 19-(R)-acetoxy-11-hydroxytabersonine (Table 1), exhibited similar antiproliferative activity against human lung adenocarcinoma cells A549, human epithelial carcinoma KB cells and P-glycoprotein (P-gp)-overexpressing MDR KB subline KB-VIN cells, with IC_50_ values ranging from 0.5 to 0.9 µM. These alkaloids efficiently induced cell cycle arrest at the G2/M phase by inhibiting tubulin polymerization as well as mitotic bipolar spindle formation. The percent inhibition of 5 µM [^3^H] colchicine binding to 1 µM tubulin in the presence of 5 µM 14,15-α-epoxy-11-methoxytabersonine (inhibition of purified tubulin assembly: EC_50_ 0.7 µM) was 54.0 ± 0.8%. Twelve benzylisoquinoline alkaloids were isolated from *Cryptocarya laevigata* [35], among which 7-hydroxylated pavine alkaloid (−)-neocaryachine (**2**, Figure 2, Table 1 and Table 2) demonstrated strong antiproliferative activity against five tested tumor cell lines, with IC_50_ values of 0.06 to 0.41 μM, including an MDR subline. Mechanism of action studies revealed that compound **2** impacted the cellular S phase by inducing DNA double-strand breaks.

Multidrug resistance mediated by the overexpression of P-gp remains the main obstacle in cancer chemotherapies. The discovery of P-gp inhibitors from natural products is a prospective strategy to combat MDR cancers. Wilforine, a sesquiterpene pyridine alkaloid found in the plant *Tripterygium wilfordii*, was found to significantly inhibit P-gp efflux via competitive inhibition, and stimulated the basal P-gp ATPase activity. In addition, wilforine re-sensitized MDR cancer cells to chemotherapeutic drugs. The docking model indicated that wilforine bound to residues of P-gp, such as LEU884, LYS887, THR176, and ASN172 [57]. A new cytotoxic C19-diterpenoid alkaloid lipojesaconitine (**3**, Table 1, Figure 2), isolated from the rhizome of *Aconitum japonicum* THUNb. subsp. *subcuneatum* (NAKAI) KADOTA, showed cytotoxicity, with IC_50_s from 6.0 to 7.3 μM against four human tumor cell lines, except a multidrug-resistant subline, suggesting that **3** was likely pumped by P-gp out of the cell [36]. It is interesting that a number of bisindole alkaloids (taburnaemine A, etc., Table 1) isolated from *Tabernaemontana corymbosa* showed antiproliferative activity (IC_50_ 2.6–9.8 μM) against several human cancer cell lines, including A-549, the triple-negative breast cancer (TNBC) cell line (MDA-MB-231, a highly metastatic cancer that lacks efficient targeted chemotherapy), ER^+^/PR^+^ breast cancer (MCF-7), KB and KB-VIN cells [37]. Camptothecin derivatives **4** and **5** (Figure 2, Table 2) were reported to display cytotoxicity against the MDR KB-VIN and parental KB tumor cell lines, while irinotecan lost its activity completely against KB-VIN [58]. In a molecular docking model, a 20-sulfonylamidine derivative of camptothecin interacted with Topo I-DNA via a different binding mode from camptothecin and irinotecan [59]. Among 7-(N-[(substituted-sulfonyl)piperazinyl]-methyl)-camptothecin derivatives, compounds **6** (IC_50_, 1.2 nM, Figure 2, Table 2) and **7** (IC_50_, 20.2 nM, Figure 2, Table 2) [60], and 10-fluorocamptothecin derivatives **8** (IC_50_, 67.0 nM, Figure 2, Table 2) and **9** (IC_50_, 99.2 nM, Figure 2, Table 2) [61], displayed potent cytotoxicity against the MDR KB-VIN cell line. 

Novel 20(S)-acylthiourea derivatives of camptothecin **10** (Figure 2, Table 2) possesses remarkable cytotoxic activity, with IC_50_s (Table 2) at the nanomolar range, being more potent than the clinical drug topotecan. Mechanistically, **10** not only induces A549 cell cycle arrest and cell apoptosis, but also inhibits Topo I activity in the cell and cell-free system in a manner similar to that of topotecan. In both xenograft and primary HCC mouse models, **10** displayed significant in vivo anticancer activity and was more potent than topotecan. In addition, there was no apparent toxicity to the liver, kidney and hemopoietic system in the FVB/N mice associated with this compound [62]. 

A novel 3,9-substituted α-carboline derivative **11** (Figure 2, Table 2) showed high levels of cytotoxicity against human leukemia cell line HL-60, colon cancer COLO 205 cell growth, Hep 3B (liver cancer cell line containing an integrated hepatitis B virus genome) and human non-small-cell lung cancer H460 cells, with IC_50_s of 0.3, 0.5, 0.7 and 0.8 µM, respectively. Mechanistic studies indicated that it targeted the G2/M phases of the cell cycle and induced apoptosis by activating death receptor and mitochondria-dependent apoptotic signaling pathways in COLO 205 cells [63].

The racemic form of isochaihulactone (Table 1), a dibenzylbutyrolactone lignan isolated from the root of *Bupleurum scorzonerifolium*, showed potent antitumor activity against A549 cells in vitro and in vivo, inhibiting A549 xenograft growth in nude mice at doses of 30 and 50 mg kg^−1^, and after structure modification, three derivatives exhibited greater activity against three tested tumor cells (A549, KB and KB-VIN) and induced cell cycle arrest in the G2/M phase [38]. Two other lignans **12** (Table 2) and **13**, isolated from the fruit of *Hernandia nymphaeifolia*, with excellent activity (IC_50_ ~5 µM) against KB-VIN cells, were reported [64], and the structures are shown in Figure 2. 

The Lee group reported that phyto-anticancer agent deoxyelephantopin derivative inhibits TNBC cell growth by inducing oxidative-stress-mediated paraptosis-like cell death [65]. Novel bis(hydroxymethyl)alkanoate curcuminoid derivative **14** (Figure 2) [66] exhibited excellent inhibitory activity against human breast cancer cell lines MCF-7 (IC_50_ 6.1 ± 0.5 µM), T47D (IC_50_ 19 ± 1 µM), HER-2^+^ breast cancer SKBR3 (IC_50_ 6.4 ± 0.4 µM), BT474 (IC_50_ 6.2 ± 0.9 µM), MDA-MB-453 (IC_50_ 4.0 ± 0.3 µM), TNBC HS-578T (IC_50_ 8.0 ± 0.3 µM), MDA-MB-157 (IC_50_ 9.2 ± 0.1 µM), MDA-MB-468 (IC_50_ 6.1 ± 0.5 µM), AR^+^ breast cancer MDA-MB-453 (IC_50_ 4.0 ± 0.3 µM), human colon cancer HCT-116 (IC_50_ 3.9 ± 0.3 µM/72 h) and classical prostate cancer PC-3 (IC_50_ 3.90 ± 0.08 µM/72 h) cell lines. It has greater inhibition than curcumin against TNBC cells and also demonstrated significant inhibition against doxorubicin-resistant MDA-MB-231 (IC_50_ 2.7 ± 0.2 µM/72 h) cells, with ten-fold higher potency than curcumin. Further, compound **14** alone was ten-fold more potent than curcumin when evaluated against the MDA-MB-231 xenograft nude mice model, and it is synergistic when used in combination with doxorubicin to treat MDA-MB-231 breast cancer cells.

**Table 2:** Selected cytotoxic compounds reported by the Lee laboratory (2016–2022) *.

Entry	Ref #	IC_50_ (µM, Unless Specified) (Average ± SD) Cell Line										
		A2780	A549	BGC-823	Hep3B	HepG-2	HCT-116	KB	KB-Vin	MCF7	MDA-MB-231	NCI-H1650
**1**	[33]	NR	0.2 ± 0.01	NR	NR	NR	NR	0.09 ± 0.02	8.9 ± 0.4	0.12 ± 0.04	0.06 ± 0.02	NR
**2**	[35]	NR	0.064	NR	NR	NR	NR	0.228	0.241	0.413	0.264	NR
**4**	[58]	NR	0.63 ± 0.02	NR	NR	NR	NR	4 ± 3	0.9 ± 0.1	2.2 ± 0.4	3.5 ± 0.5	NR
**5**	[58]	NR	0.60 ± 0.03	NR	NR	NR	NR	5 ± 4	0.38 ± 0.08	4.7 ± 0.5	5.4 ± 0.5	NR
**6**	[60]	NR	5.4 ± 0.2 nM	NR	NR	NR	NR	21 ± 5 nM	0.14 ± 0.03	0.07 ± 0.01	0.12 ± 0.02	NR
**7**	[60]	NR	6.9 ± 0.8 nM	NR	NR	NR	NR	0.10 ± 0.05	20 ± 1 nM	35 ± 8 nM	0.08 ± 0.01	NR
**8**	[61]	NR	9.9 ± 0.4 nM	NR	NR	NR	NR	70 ± 2 nM	67.0 ± 0.8	77 ± 5 nM	0.3 ± 0.1	NR
**9**	[61]	NR	8.72 ± 0.03 nM	NR	NR	NR	NR	47 ± 6 nM	0.10 ± 0.02	81 ± 4 nM	0.24 ± 0.03	NR
**10**	[62]	NR	4 ± 3 nM	NR	2.3 ± 0.0 nM	NR	NR	24 ± 9 mM	0.05 ± 0.04	42 ± 1 nM	NR	NR
**11**	[63]	NR	8.1	NR	NR	NR	NR	20.3	5.4	6.8	20.8	NR
**12**	[64]	NR	5.7	NR	NR	NR	NR	12.6	5.3	8.1	8.2	NR
**15**	[67]	1.12	NR	4.1 ± 0.0	NT	2.28	0.76	NR	NR	NR	NR	1.2 ± 0.0
**16**	[67]	2.08	NR	9.4 ± 0.0	NT	2.11	0.86	NR	NR	NR	NR	1.3 ± 0.0
**17**	[67]	2.03	NR	2.7 ± 0.0	NT	3.03	2.15	NR	NR	NR	NR	1.6 ± 0.0
**18**	[68]	NR	6.1 ± 0.4	NR	NR	NR	NR	6.5 ± 0.3	7.0 ± 0.6	9.2 ± 0.9	11.0 ± 0.9	NR
**18a**	[68]	NR	5.6 ± 0.4	NR	NR	NR	NR	6.7 ± 0.5	6.7 ± 0.0	15.3 ± 0.6	9.0 ± 0.2	NR
**18b**	[68]	NR	4.5 ± 0.3	NR	NR	NR	NR	6.1 ± 0.5	6.0 ± 0.3	14.4 ± 1.1	12.4 ± 0.3	NR
**19**	[69]	NR	0.4 ± 0.1	NR	NR	NR	NR	0.53 ± 0.03	0.8 ± 0.3	1.0 ± 0.2	0.59 ± 0.06	NR

* IC_50_: the concentration of compound that caused a 50% reduction relative to untreated cells determined by the SRB assay. SD: standard deviation. NR: not reported in the original paper. A2780 (human ovarian cancer), A549 (lung cancer), BGC-823 (human gastric cancer cell line), Hep3B (hepatocellular carcinoma, HCC), HepG-2 (liver hepatocellular cells), HCT-116 (human colon cancer), KB (nasopharyngeal carcinoma), KB-vin (vincristine resistant KB subline), MCF (Michigan Cancer Foundation-7), MDA-MB-231(human breast carcinoma cell line), NCI-H1650 (non-small-cell carcinoma) [33,35,58,60,61,62,63,64,67,68,69].

Novel indolin-2-ones (Table 2, Figure 2) **15** and **16** are potent inhibitors of HCT-116 cells. Moreover, **15** and **17** are also potent inhibitors of TNBC cell line MDA-MB-231. Flow cytometry was utilized to explore the antitumor mechanism of **15** and **17**, and distinct effects were observed on **17**. Further, immunocytochemical examination of **15** suggested the destabilization of microtubules [67].

Betulinic acid (BA), a pentacyclic triterpenoid, generally exhibits modest antiproliferative activity. To improve its potency, one fluorine atom was introduced at C-2, creating two diastereomers. It is interesting that a racemic 2-F-BA (**18**, a mixture of **18a**/**18b**, Figure 2, Table 2) [68] showed significantly improved antiproliferative activity, while each diastereomer exhibited similar effects. Moreover, 2-F-BA was found to be a topoisomerase (Topo) I and IIα dual inhibitor in cell-based and cell-free assays. A hypothetical mode of binding to the Topo I-DNA suggested a difference between the hydrogen bonding of BA and 2-F-BA to DNA.

The antitumor effects of the natural product piperlongumine appear to result from increasing intracellular ROS levels via the inhibition of antioxidative thioredoxin reductase (TrxR). After structure modification, an analog **19** (Figure 2, Table 2) showed 12-fold higher inhibitory activity with low acute toxicity [69].

New dammarane-type saponins isolated from *Gynostemma pentaphyllum* were found to be quite active against two human tumor cell lines (A549 and human liver cancer cell line HepG2), with an EC_50_ range of 29–60 µM [70]. The strong hemolytic adverse effect of pulsatilla saponin D, isolated from *Pulsatilla chinensis* (Bunge) Regel (Ranunculaceae) and other related plants, has hampered its clinical development as an injectable anticancer agent. With ring C, C-28 or C-3 modifications, the hemolytic activity of 17 derivatives dropped dramatically [71], and compound **20** (Figure 2) exhibited cytotoxicity toward A549 (IC_50_ 2.8 ± 0.5 μM) in a dose-dependent manner, being more potent than the parent compound pulsatilla saponin D (IC_50_ 6.0 ± 0.7 μM).

### 2.2. Antiviral Agents

Viral infections affect millions of people annually, and these infections have raised serious concerns regarding global public health. Due to the limited efficacy and serious adverse effects associated with commonly used antivirals and the growing incidences, especially of resistant viral strains, the available therapeutic modalities need to be improved and complemented by novel antiviral agents [72]. Plants have been used to treat viral infections for a long time. Akram et al. focused on the therapeutic potential of 54 medicinal plants (belonging to 36 families, pure compounds excluded) in the eradication and management of various viral diseases, such as influenza, human immunodeficiency virus (HIV), herpes simplex virus (HSV), hepatitis, and coxsackievirus infections [72]. Various antiviral natural-product-related agents have been discovered from natural resources during the past several decades. Flavonoids [73], terpenoids, alkaloids, and other widely distributed plant secondary metabolites have been reported to exhibit significant antiviral properties in in vitro and in vivo studies. 

Hepatitis B virus (HBV) remains a significant health threat and has a lifetime impact on living quality for those who are infected. Three alkaloids composed of fused quinolizidine and octahydroquinoline rings, ochrocephalamines B-D, isolated from *Oxytropis ochrocephala* Bunge, demonstrated potent anti-HBV activities and were reported to be more potent against the secretion of HBeAg than that of HBsAg [39]. 

HIV is the pathogen that causes acquired immune deficiency syndrome (AIDS, the most advanced stage of the infection). According to the World Health Organization (WHO) [74], around 650,000 people died from HIV-related diseases and around 1.5 million people acquired HIV in 2021. There are an estimated 38.4 million people living with HIV currently, two-thirds of whom live in the WHO African Region. There is no cure for HIV infection, but treatment and care may enable people with HIV to live a long life. The U.S. FDA-approved clinically used drugs for AIDS patients include eight categories: entry or fusion inhibitors, nucleoside or nucleotide or non-nucleoside reverse transcriptase inhibitors, HIV protease inhibitors, HIV integrase strand transfer inhibitors, and multi-class combination products. The major problems associated with the treatments are drug toxicity and resistance developed over time. HIV patients are usually placed on a minimum of three drugs for combination therapy. To overcome drug resistance, natural product/medicinal chemists have conducted intensive bioassay-directed isolation and structure modifications of natural products. The Lee laboratory had a number of publications on pure, potent anti-HIV agents isolated from natural resources, some of which are promising leads for future drug development. Representative compound antiviral data, especially against drug-resistant HIV mutant strains, are listed in Table 3, and compound structures are shown in Figure 3. 

A new carbazole alkaloid (**21**, Figure 3) isolated from *Clausena anisumolens* was reported to have anti-HIV activity, with an EC_50_ of 2.4 µg/mL and SI of 7.1 in MT-4 lymphocytes infected by HIV-1NL4-3 Nanoluc-sec virus [75]. Diarylnicotinamide derivatives (Figure 3, Table 3) propionitrile (**22**), pyrrolidin-1-ylmethanone (**23**) and morpholinomethanone (**24**), the HIV-1 non-nucleoside reverse transcriptase inhibitors (NNRTIs), showed good to excellent activity against a wild-type HIV-1 strain, with EC_50_s of 0.02–1.77 µM, and are equally potent towards the clinical drug etravirine against a HIV-1 E138K mutant strain, but with much lower cytotoxicity [76]. A quinolizidine-type alkaloid aloperine, initially isolated from the seeds and leaves of *Sophora alopecuroides* L., was found to block HIV-1 entry. It inhibited HIV-1 envelope-mediated cell–cell fusion at low micromolar concentrations. One derivative (**25**, Figure 3) with an N-(1-butyl)-4-trifluoromethoxybenzamide side chain showed anti-HIV-1 activity, with an EC_50_ at 0.69 μM. These compounds are a new class of anti-HIV-1 entry inhibitors [77]. 

Diterpenes isolated from the families Thymelaeaceae and Euphorbiaceae are grouped into three structural types—daphnane, ingenane and tigliane—which exhibited significant anti-HIV activity [78,79]. A number of potent anti-HIV tigliane diterpenoids from *Reutealis trisperma* [80] (IC_50_s 2.30–4.03 μM) and *Wikstroemia lamatsoensis* [81,82] (IC_50_s 0.18–12.80 nM) have been reported. Recently, nine potent anti-HIV daphnane diterpenoid orthoesters (**26**–**34**, Table 3) (**30**, Figure 3) [83] and novel gnidimacrin-related macrocyclic daphnanes, daphneodorins A and B (**35**, **36**, Figure 3, Table 1 and Table 3) [40], isolated from *Daphne odora* with EC_50_ values of 0.16–7.70 nM were reported. Potent anti-HIV daphnane diterpenes were also isolated from petroleum ether extract of *Daphne genkwa* [84]. Structure modification of ingenane esters isolated from *Euphorbia kansui* produced a potent anti-HIV-1 derivative, 3-(2-naphthoyl) ingenol (**37**, Figure 3), with an IC_50_ value of 1.3 ± 0.5 nM [85]. It also acted as an HIV-1 latency-reversing agent, activating HIV-1 replication in a latently infected U1 cell model and a T cell latent HIV-1 model, JLat-A2. Another example of a compound that activates latent HIV-1 replication and inhibits HIV-1 infection is gnidimacrin (at picomolar concentrations), and several of its derivatives were also reported as potent HIV-1 Inhibitors and HIV latency-reversing agents [86].

Certain triterpenes and their analogs have demonstrated potential as pharmaceutical precursors for the treatment of HIV [87]. Several antitumor/HIV triterpenoids have been isolated from the methanol extract of *Kleinhovia hospital*, among which kleinhospitine E (Table 1) was reported as the first cycloartane triterpenoid alkaloid possessing an unusual γ-lactam with an oxopropylidene side chain [41]. Novel betulinic/betulonic acid–nucleoside hybrids were found to be highly effective against HIV [88]. After bioassay-directed isolation, pure triterpenoid beesioside (Table 1) from the roots of *Souliea vaginata* showed anti-HIV activity with an EC_50_ of 2.32 µM (CC_50_ > 40 µM). Further structural modification gave compound **38** (Figure 3, Table 3), with an EC_50_ value of 0.025 µM and TI value greater than 800 [42]. 

**Table 3:** Representative compounds’ activity against MT-4 cells infected with HIV-1 wild and mutant strains, as reported by the Lee laboratory (2016–2021) *.

Entry	Ref #	EC_50_ (µM)									SI		IC_50_ (nM)	CC_50_ (µM)
		E138K	IIIB	K103 N	L100I	NL4-3 (nM)	RES056	Y181C	Y188L	F227L + V106A	IIIB	RES056		
**22**	[76]	0.015 ± 0.003	0.020 ± 0.005	0.25 ± 0.03	1.1 ± 0.2	NR	2.3 ± 0.6	0.089 ± 0.004	2.24 ± 0.07	0.3 ± 0.2	2044	17	NR	40.15
**23**	[76]	0.014 ± 0.001	0.020 ± 0.005	0.270 ± 0.006	1.0 ± 0.1	NR	1.4 ± 0.3	0.09 ± 0.02	1.48 ± 0.08	0.240 ± 0.008	2897	41	NR	58.09
**24**	[76]	0.027 ± 0.001	0.020 ± 0.009	0.38 ± 0.05	6.1 ± 0.2	NR	16 ± 7	0.24 ± 0.09	7.2 ± 0.5	0.610 ± 0.007	9279	12	NR	180.9
**26**	[83]	NR	NR	NR	NR	8 ± 2	NR	NR	NR	NR	NR	NR	>34	NR
**27**	[83]	NR	NR	NR	NR	1.5 ± 0.4	NR	NR	NR	NR	NR	NR	>34	NR
**28**	[83]	NR	NR	NR	NR	2.2 ± 0.8	NR	NR	NR	NR	NR	NR	>34	NR
**29**	[83]	NR	NR	NR	NR	1.9 ± 0.4	NR	NR	NR	NR	NR	NR	>34	NR
**30**	[83]	NR	NR	NR	NR	1.6 ± 0.5	NR	NR	NR	NR	NR	NR	>34	NR
**31**	[83]	NR	NR	NR	NR	5 ± 1	NR	NR	NR	NR	NR	NR	>34	NR
**32**	[83]	NR	NR	NR	NR	4 ± 1	NR	NR	NR	NR	NR	NR	>34	NR
**33**	[83]	NR	NR	NR	NR	1.9 ± 0.5	NR	NR	NR	NR	NR	NR	>34	NR
**34**	[83]	NR	NR	NR	NR	2.3 ± 0.5	NR	NR	NR	NR	NR	NR	>34	NR
**35**	[40]	NR	NR	NR	NR	0.16 ± 0.06	NR	NR	NR	NR	NR	NR	>25	NR
**36**	[40]	NR	NR	NR	NR	0.25 ± 0.06	NR	NR	NR	NR	NR	NR	>25	NR
**38**	[42]	NR	NR	NR	NR	25 ± 9.5	NR	NR	NR	NR	NR	NR	NR	>20

* EC_50_: concentration of compound required to achieve 50% protection of MT-4 cell cultures against HIV-1-induced cytotoxicity, as determined by the MTT method. CC_50_: concentration required to reduce the viability of mock-infected cell cultures by 50%, as determined by the MTT method. SI: selectivity index, the ratio of CC_50_/EC_50_. NR: not reported in the original paper [40,42,76,83].

The world is currently experiencing a novel COVID-19 pandemic caused by severe acute respiratory syndrome coronavirus (SARS-CoV-2). As of 30 October 2022, 627 million confirmed cases and 6.5 million deaths have been reported globally [89]. To date, available therapeutic options are still very limited. Herbs, natural drugs or related analogs are important alternative therapies in the prevention, treatment, and recovery from the infection. The mortality rate was found to be lower in patients treated with oseltamivir, an anti-influenza drug synthesized using two natural products, quinic acid and shikimic acid, than in the control group (1.7% vs. 6.7%, *p* = 0.06), and the administration of oseltamivir was associated with a shorter length of hospital stay and earlier recovery/discharge from hospital [90]. A number of natural compounds have proven to have anti-human coronavirus activities [91,92,93], and certain combinations of herbs are effective to prevent the infection and relieve the associated symptoms, but further research on natural products to identify more potent new leads is urgently needed. Su et al. recently reported their discovery of myricetin (**39**, Table 1, Figure 4), a flavonoid in many foods, as a non-peptidomimetic and covalent inhibitor (IC_50_ = 0.63 µM) of the SARS-CoV-2 3-chymotrypsin-like cysteine protease (3CL_pro_), a highly conserved cysteine proteinase required for coronaviral replication. Co-crystal structures of the protease bound with myricetin and its derivatives revealed that the pyrogallol group worked as an electrophile to covalently modify the catalytic cysteine, which demonstrated that the pyrogallol can serve as an alternative tool in the design of targeted covalent ligands [43]. Another example of natural products against a SARS-CoV-2 enzyme was reported by Du et al. [44]. They discovered that both polyphenols (Table 1, Figure 4) chebulagic acid (**40**, originally isolated from the fruit of *Terminalia chebula*) and punicalagin (**41**, found in pomegranates) reduced SARS-CoV-2 virus-induced plaque formation in a Vero-E6 monolayer at noncytotoxic concentrations, with IC_50_ values of 9.09 ± 0.87 μM and 4.62 ± 0.27 μM, respectively, by targeting the viral 3CL^pro^ as allosteric regulators.

Herpes simplex virus (HSV) belongs to Herpesviridae, which is a broad family of enveloped DNA viruses that induce numerous clinically substantial syndromes in both adults and neonates. There is a strong association between HSV type 2 (HSV-2) and HIV infection. In 2017, a systematic review and meta-analysis of 55 prospective studies found that the risk of becoming infected with HIV was at least tripled in people with HSV-2 [94]. Recently, Treml et al. [95] conducted a review on certain natural antiherpetic agents. According to their review, the incorporation of safe and effective natural products into anti-HSV drug development has gained limited attention to date, and the recent dramatic increase in HSV-1 (HSV type 1) and HSV-2 has caused a significant challenge to public health due to drug resistance. 

### 2.3. Antibacterial Drug Discovery

It is estimated that approximately 50% of the antibacterial drugs approved by the U.S. FDA between 1981 and 2019 were based on molecules obtained from natural resources. Most drugs in the “golden age” of discovery of antibacterial agents (1940–1962) [96,97,98], including drug classes such as penicillins and aminoglycosides, were based on natural resources [98]. While penicillin was discovered in fungi, the majority of antibacterial drugs from natural resources were based on molecules from prokaryotic microorganisms [98], and specifically terrestrial actinomycetes [32,97,99]. The medicinal chemistry age of antibacterial drug discovery (1950–1980) was distinguished by chemical modifications mostly from drug classes that were discovered earlier [96,97,100]. From the 1980s to the 2010s, the frequency of approval of new antibacterial medications in the U.S. was reduced from 61 to 34 per decade, respectively [101]. This decrease was attributed to various factors, such as the reduced profitability of antibacterial drugs [102]. At the same time, bacterial resistance to drugs became an even more severe healthcare problem [97,103,104,105]. Efforts were conducted by regulatory agencies and the U.S. government through Medicare to enhance the financial appeal of antibacterial medications [99,106,107]. This was conducted to enhance the discovery of additional antibacterial medications [97,108], including drugs that are effective against drug-resistant bacteria [98].

The classical approach for the discovery of antibacterial drugs was based on the screening of a large number of synthetic or semisynthetic molecules for their antibacterial activity [103]. With the advent of techniques involving genomics and proteomics, greater efforts were conducted to screen molecules against specific bacterial targets that may be susceptible to drugs and meet additional requirements such as being different from eukaryotes [96,99,103].

Notable small-molecule antibacterial drugs based on natural resources and approved worldwide since 2016 are plamozicin, omadacycline, eravacycline, sarecycline, lefamulin, and cefiderocol. At the same time, Vabomere and Recarbrio, products that combine antibiotic adjuvants with an antibacterial agent, were approved for clinical use. Both products contain a β-lactamase inhibitor and a carbapenem β-lactam antibacterial agent related to a natural resource [109,110].

Recarbrio is a combination of imipenem, a carbopenem, relebactam (**42**, Figure 5), a β-lactamase inhibitor, and an inhibitor of the renal enzyme dehydropeptidase I, cilastatin [109,111]. Chemically, imipenem is an N-formimidoyl thienamycin [111,112] and, following its discovery, it was shown to be stable to β-lactamase. Hence, prior to the approval of Recarbrio, the combination of imipenem with cilastatin was marketed in the U.S. without a β-lactamase inhibitor as Primaxin IV. In 2008, bacterial resistance led to renewed efforts to discover β-lactamase inhibitors [101]. One such agent is relebactam, a diazabicyclooctanone analog [113] that is one of the components of Recarbrio.

Vabomere is a combination of a carbapenem drug, meropenem, and a β-lactamase inhibitor, vaborbactam. Meropenem was developed as part of an effort to design carbapenems that are more stable and have a broader antibacterial spectrum. The addition of a pyrrolidine side chain broadened its antibacterial spectrum. Methylation enhanced its stability to the human dehydropeptidase I [112]. Hence, there was no need for an inhibitor such as cilastatin [109]. Vaborbactam is the first-in-class boronic acid β-lactamase inhibitor that was developed [110,113].

Cefiderocol is a cephalosporin antibacterial agent, which contains a catechol moiety that provides siderophore activity [105]. A common definition of a siderophore is “any of a group of low molecular weight compounds produced especially by various microorganisms that bind ferric iron extracellularly to form a stable chelate for transport into the cell”. The unique conjugation of a catechol moiety to the structure enabled the antibacterial agent to form a complex with iron and to enter the bacterial cell through an iron transporter. This enhanced the uptake of the drug into bacterial cells. Moreover, a pyrrolidinium group confers stability against a wide variety of common β-lactamases [105]. The drug is effective against some critical drug-resistant bacteria [105,109,114].

Lefamulin is a semisynthetic antibacterial agent from the pleuromutilin class. The class was discovered in 1951 [115] and it is based on tricyclic diterpenoid pleuromutilin obtained from an edible mushroom [104]. Other compounds from the class had veterinary applications or were used to treat topical infections in humans (retapamulin) [115]. Lefamulin is the first antibacterial agent from the pleuromutilin class approved for systemic uses in humans [115], and it has lower toxicity compared to other drugs that treat community-acquired bacterial pneumonia [116].

Omadacycline is a tetracycline antibacterial agent based on minocycline that has a favorable oral absorption profile [117]. Tetracyclines were originally obtained from bacteria [118]. Omadacycline was designed to be well absorbed, overcome tetracycline resistance [119] and avoid gastrointestinal disturbances that appeared in other recent tetracycline antibacterial agents, such as glycylcyclines, including eravacycline. A reactive intermediate, 9-aminomethylminocycline 4, that can relatively easily be synthesized from minocycline accomplished these goals and was named omadacycline. This compound was superior to other aminomethylcyclines and exhibited a wide antibacterial spectrum [117].

Studies are ongoing to develop new antibacterial agents, including those that may be effective against pathogens from the WHO priority pathogen list [109]. An example of a new class of antibacterial agents is the arylomycins that are based on metabolites isolated from Streptomycete, a strain of terrestrial actinomycetes [120,121]. G0775, an agent from this class, exhibited activity against drug-resistant bacteria [121]. A different example of a new antibacterial agent from a natural resource is ubonodin, a lasso peptide [45]. This agent was discovered by the genome mining [97,99] of a microorganism, *Burkholderia ubonensis* MSMB2207. It has an atypically large size of 28 amino acids and demonstrated antibacterial activity against members of the *Burkholderia cepacia* complex [45]. Examples of novel antibacterial agents are complestatin and corbomycin (**43**, Figure 5). These agents were discovered by a unique procedure of gene sequencing of actinomycetes. The novel mechanism of action of the agents includes the inhibition of peptidoglycan modeling and the agents are active against drug-resistant bacteria [122]. Chemical structures of the above-described drugs are shown in Figure 5.

Revitalization of antibacterial drug discovery is expected to include the discovery of drugs from various resources [106]. It is possible that even in an age of bacterial resistance, there are still a variety of antibacterial agents from natural resources that may be effective and have not yet been identified [99,103,123]. Hence, drug discovery for antibacterial agents may include, for example, the identification of antibacterial molecules from natural resources that were less studied, including those that grow in more extreme conditions such as desert soil or deep oceans [97,103].

### 2.4. Antimalarial Drugs

Humans have long used plants as sources of medicine. Some of the earliest records come from Sumerian clay tablets dated 2600 BC [124]. Newman and Gordon have published multiple reviews that have demonstrated that natural products still play a major role in the discovery of drug development leads to combat human diseases [125,126,127]. Malaria and other protozoan parasitic diseases occur mainly in underprivileged populations in developing tropical and subtropical regions of Asia, Africa, and the Americas. According to the 2021 WHO World Malaria Report, there were an estimated 241 million malaria cases in 2020 in 85 malaria-endemic countries (including the territory of French Guiana), increasing from 227 million in 2019, with most of this increase coming from countries in the WHO African Region [128]. The increase in 2020 was associated with the disruption of services during the COVID-19 pandemic. This report stated that 163 million cases and 444,600 deaths were found in HBHI (high burden to high impact) countries in 2020 [128]. Malaria is caused by a protozoan parasite in the genus *Plasmodium*. There are five identified species that can infect humans. *P. falciparum* (endemic to Africa) and *P. vivax* (endemic to the Americas) are responsible for 95% of malaria cases [129]. *Plasmodium falciparum* has been identified as the most lethal because it produces cerebral malaria infections [128]. Quinine has long been the standard treatment for malaria. Spanish colonists in Peru during the 1500s found that drinking extracts from Cinchona bark cured their malarial symptoms [130]. Quinine is still an important treatment for malaria; however, resistance has slowly developed and quinine itself has a low therapeutic index due to significant side effects [130]. Dr. Youyou Tu and a group of Chinese scientists discovered artemisinin to combat malaria in 1971, and this drug has saved millions of lives [131,132,133]. However, artemisinin-resistant strains have been reported in Cambodia and in the African Continent. According to the Center for Disease Control, both *P. falciparum* and *P. vivax* have developed resistance to antimalarial drugs. *P. falciparum* in particular has developed resistance to sulfadoxine/pyrimethamine, mefloquine, halofantrine, quinine, artemisinin and non-artemisinin components of artemisinin-based combination therapies [134]. Therefore, it is imperative to find other antimalarial compounds to avoid increased suffering and death due to malaria, and natural products and their derivatives afford a variety of potential leads. An excellent review of antimalarials from natural sources can be found in Rahul Jain’s publication in *Bioorganic and Medicinal Chemistry* in 2009 [135]. This discussion will look at recent frontrunners to treat malaria that have been found in a variety of natural sources and have structures that do not resemble quinine’s or artemisinin’s benzopyridine and endoperoxide 1,2,4-trioxane ring systems, respectively, to help avoid resistance issues. 

Mennezes and Campos recently published a review examining natural bioflavonoids as antimicrobial treatments [136]. They found that dimeric flavonoids are more effective than monomeric ones in the treatment of a variety of microbial diseases. Four publications were highlighted in this review for dimeric flavonoids that showed antimalarial activity. Lanaroflavone, which has many phenol groups, had an IC_50_ = 0.48 µM and a selectivity index of 159 on L6 cells against K1 strains of *P. falciparum* [137]. K1 strains of this organism are often studied as they have MDR; FCR3 is resistant to chloroquine, and 3D7 strains do not have resistance to drugs [129]. Heveaflavone (7,4′,7″-tri-O-methylamento-flavone), with three phenol and three methyl substituents, had an IC_50_ = 0.26 µM and was also active against K1 strains of *P. falciparum* [138]. Methylenebissantin, with multiple phenol and methoxy groups, had an IC_50_ = 0.91 µM and reduced enoyl-ACP reductase activity against *P. falciparum* [139]. GB1 (3″,4′,4,5,5″,7,7″-heptahydroxy-3,8-biflavanone), isolated from *Garcinia kola* stem bark, had an IC_50_ = 0.16 µM, an SI > 900 and was also active against *P. falciparum* [140].

Another recent review by Justus Nweze examined natural marine products as potential antimalarial drugs [141]. The article listed 125 compounds that displayed activity against malaria, leishmaniasis, and trypanosomiasis parasites that included alkaloids, terpenoids, peptides, polyketides, terpenes, coumarins, steroids, fatty acid derivatives, and lactones. This review focuses on the findings that displayed an effect on strains of *P. falciparum*. Parra reported that bromopyrrole alkaloids from the marine sponge *Tedania brasiliensis* could serve as new platforms for novel antimalarial compounds [142]. Pseudoceratidine is a secondary amine with mirroring butyl chains connected to an amide linkage to terminal brominated pyrrole rings. This compound was highly potent against 3D7 and K1 strains, with IC_50_ = 0.96–1.24 µM, as were many natural and synthetic derivatives. A novel linear diterpene with two stereogenic centers, bifucatriol (**44**, Figure 6), was found to inhibit *P. falciparum* K1 at low concentrations, IC_50_ = 0.65 µg/mL. It was isolated from Irish brown alga (*Bifurcaria bifurcate*) and could become a lead compound with modifications, as it was slightly toxic to RSM L6 cell lines (56.6 µg/mL) [46]. Another study found three sesquiterpenoids, smenotronic acid, ilimaquinone and pelorol, to have strong in vitro activity against a chlorine-resistant *P. falciparum* Dd2, with IC_50_ values of 3.51, 2.11 and 0.8 µM. These compounds were isolated from the marine sponge *Hyritios erectus* [143]. Kakeromamide (**45**, Figure 6), a novel cyclic peptide, as well as fellow macrocycles, ulongamide A and lungbyabelline A, were obtained from the marine cyanobacterium *Moorea producens*. These inhibited the blood stages of *P. falciparum* with EC_50_s values of 8.9, 0.99 and 1.5 µM. Sweeney-Jones proposed that kakeromamide B interacts with actin-like proteins and a sortilin protein that disrupts the invasion of host cells. The compounds also gave low cytotoxicity levels of >23, >31 and >12 µM on HEK293T and >23, 17 and >13 µM on HepG2 cell lines [47]. A forty-atom macrocycle was isolated from cyanobacterium *Okennia hirsute* and found to display high anti-plasmodial activity against the *P. Falciparum* HB3 strain, which is known to be sensitive to chloroquinone [144]. Several sterols have been discovered with interesting antimalarial properties. A novel mono-hydroxy acetylated sterol derivative, halymeniaol (**46**, Figure 6), showed selectivity towards 3D7 strains with an IC_50_ = 3.0 µM. This compound was collected from a marine alga, *Halymenia floresii*, and was not cytotoxic to N2A and RAW 264.7 cells [48]. Extracts from the marine sponge *Xesto spongia* produced two sterols with potent antimalarial responses to 3D7 strains. Kaimanol and sarignosterol have an IC_50_ of 359 and 0.250 nM, respectively. The publication also speculated that a benzoyl group decreases the activity and, conversely, an olefinic substituent will increase the antimalarial responses of sterols [145].

Other alkaloid structures beyond quinoline have been shown to be active against malaria. Two indole alkaloids with a 6H-pyrido [4,3-b]carbazole backbone have shown potent activity. Ellipticine was first isolated from an Australian tree, *Ochrosia elliptica*, and has IC_50_ values of 0.81 µM and 0.5 µM, respectively, against the KI and 3D7 strains of *P. falciparum*. Olivacine is a structural isomer of ellipticine and has IC_50_ values of 0.14 µM and 1.2 µM, respectively, against the KI and 3D7 strains. These alkaloids demonstrated that they were not highly toxic to murine macrophages, with an SI of >500 for ellipticine and >290 for olivacine [146]. Violacein is a violet pigment that was isolated from *Chromobacterium* sp. but is now available from *E. coli* by synthetic biology [147]. This indolocarbazole alkaloid is active against 3D7 strains, with an IC_50_ value of 0.51 µM. Importantly, it has also been shown to be effective against artemisinin-sensitive (ANLI) and artemisinin-resistant (APL5G) strains of *P. falciparum*, with IC_50_ values of 0.74 µM and 0.76 µM [148]. Moreover, 7-chloroviolacein is a biosynthetic analog that places a chlorine atom at the seventh position in the indolin-2-one ring system. It is more active than violacein, with an IC_50_ value of 0.42 µM. These compounds could provide promising antimalarial leads as violacein has a new mode of action against the parasite because it affects the actin cytoskeleton [149].

This short review of recent publications on antimalarial natural products has indicated that a number of potent drugs could be obtained from structural motifs that are different from quinine and artemisinin. This provides hope for the possibility of several different strategies to provide compounds that may offer agents that will be active against even *P. falciparum*-resistant malarial parasites.

### 2.5. Marine Bioactive Natural Products

As stated earlier in this review, many natural pharmaceutical products come from marine sources. This resource will continue to be a crucial source of medicines for future generations with continued work in the field. The variety of diseases targeted by natural marine products is impressive, and a number of them will be discussed here.

Given the sudden need for anti-COVID-19 medicines, Pawar, Dimri, Maithani and Kush [49] reviewed a number of structures related to anti-COVID-19 (SARS-CoV-2) activity. They focused on 3CL^pro^ inhibitors and found targets through screening chemical libraries. Their methodology involved comparing antiviral potencies using virtual screening, homology modeling and molecular docking. The docking scores and binding affinities (kcal/mol) were compared between twelve of the top candidates, with 5,7,3′4′-tetrahydroxy-2′-(3,3-dimethylallyl)isoflavone (**47**, Figure 7) being the top-scoring phytochemical, with a docking score of −16.35 and a binding affinity of −29.57 kcal/mol.

A brief review in 2021 by Taglialatela-Scafati [150] of natural marine products discussed some recent drug candidates that were repurposed from other applications. They highlighted marine cyanobacterial depsipeptide gallinamide A (**48**, Figure 7) and its synthetic analogs as potent inhibitors of cathepsin L, which is used by coronaviruses to release RNA material into cells [50]. Another exciting candidate with anti-COVID-19 potency is another depsipeptide, plitidepsin, which is derived from the tunicate *Aplidium albicans* [151]. The article, published in Science, describes how plitidepsin outperformed the now famous drug, remdesivir, against SARS-CoV-2. Plitidepsin, originally prescribed for myeloma and marketed under the name Aplidin^®^, targets the human protein eEF1A, which is involved in cancer insurgence, and also greatly reduces the viral load and lung inflammation during SARS-CoV-2 infection.

A review by Tan and Phyo [152] focused on marine cyanobacteria and their production of particularly potent molecular therapeutic agents. The review was presented according to the types of targets, including both enzymatic and non-enzymatic, and described specific diseases with multiple derivatives of each class of compounds. For example, largazole is used in many applications, as it is a very potent class I histone deacetylease (HDAC) inhibitor [153]. Another HDAC inhibitor class, santacruzamate A (**49**, Figure 7), and the similar suberoylanilide hydroxamic acid (SAHA) were reviewed and highlighted for the fact that santacruzamate A is a potent inhibitor of HDAC2, with an IC_50_ of 119 pM [51]. Another class of natural products derived from marine cyanobacteria target proteasomes, with carmaphycin A and B being the focus of this class. These proteasome inhibitors have IC_50_ values in the 2.5 nM range, and are also effective against solid tumor lines [154]. Serine protease inhibitors were also discussed, which marine cyanobacteria may use as a chemical defense system against predators. Many of these compounds are 3-amino-6-hydroxypiperidone (Ahp) cyclodepsipeptides. These serine protease inhibitors, such as the lyngbyastatins and tutuilamides, for example, have roles related to human health, such as in the immune response. Again, gallinamide A was discussed as a potent antiviral compound, and it acts as a falcipain inhibitor, which targets cysteine proteases. It has an EC_50_ of 74 nM against *Plasmodium falciparum* [155]. Cathepsins are another class of protease enzymes with a wide range of functions in humans, which makes these enzymes good targets for a number of therapeutic agents. More depsipeptides, such as grassystatins, tasiamides and symplocin A, were found to be potent cathepsin inhibitors. For example, grassystatins A and B displayed IC_50_ measurements of 16.9 nM and 0.62 nM, respectively, against a class of cathepsins [156]. Tasiamides B and F displayed IC_50_ values of 50 nM and 9.0 nM for the same cathespins [157]. Tasiamide B is also a β-secretase 1 (BACE1) inhibitor, with an IC_50_ value of 0.19 uM against BACE1 [158], has the potential to target Alzheimer’s disease, as BACE1 is involved in abnormal β-amyloid plaque production. Apratoxin A and coibamide A are depsipeptides that both target Sec61 protein translation channels, which leads to a number of applications, especially in targeting cancer cells by binding to proteins and thus directly blocking the protein translation channel, sec61α [159]. Finally, Tan and Phyo discussed honaucins, which are derived from *Leptolyngbya crossbyana* and are potent anti-inflammatory and bacterial quorum-sensing inhibitory molecules [160]. A relatively simple molecule when compared to the many depsipeptides, honaucin A (**50**, Figure 7) showed the ability to attenuate inflammation through the activation of the Nrf2-ARE pathway [52].

## 3. Computational Methodology for Natural-Product-Based Drug Discovery

Chemical drugs typically work by interacting with a specific target involved in the pathology of a disease, which underlies the mechanism of drug action. In a typical drug discovery campaign, high-throughput screening (HTS) technologies are frequently employed based on well-developed cell-free or cell-based assays established for the implicated target or pathway. Virtual screening techniques are often used based on either computational ligand-based drug discovery (LBDD) approaches or structure-based drug discovery (SBDD) methods, depending on the availability of the target 3D structure and/or structure–activity relationship (SAR) data [161]. In more recent years, emerging technologies such as literature-wide association studies (LWAS) and network-wide association studies (NWAS) have been proposed. The former is based on text mining analyses of scientific publications available in PubMed [162], and the latter on large drug-disease-target (DDT) network graph data being constructed (such as ROBOKOP [163]). Here, we review both the traditional methods and their selected applications to natural product research and those conducted by K.-H. Lee’s laboratory, as well as the emerging technologies: text mining for drug repurposing and knowledge-graph-based methods for drug repurposing.

### 3.1. Structure-Based Drug Discovery (SBDD)

**Figure 8:**
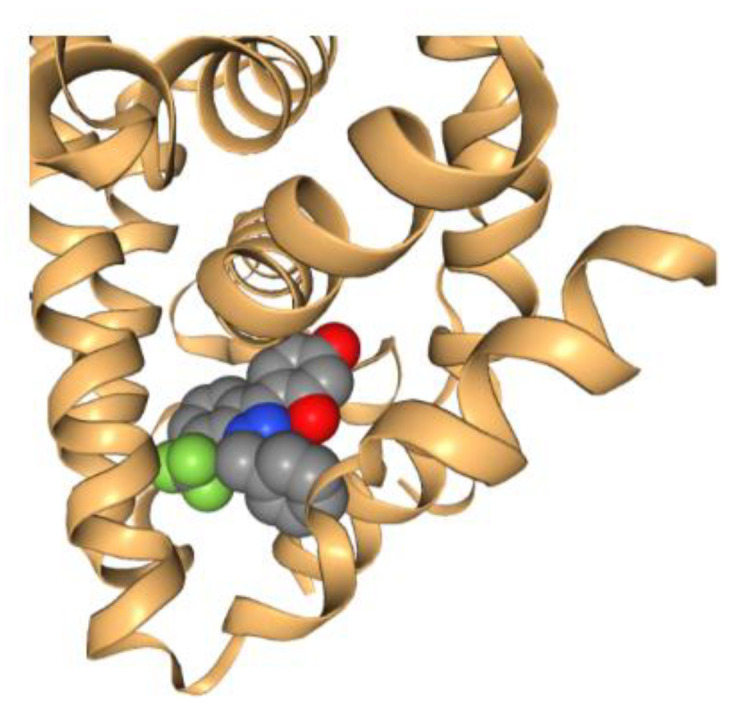
Estrogen receptor structure (3OS8) bound with a ligand, showing the basis of complementarity for ligand–receptor docking.

Since the pioneering work of Perutz and Kendrew [164,165] that earned them the Nobel Prize in Chemistry in 1962, the use of crystallography in solving the X-ray crystal structures of proteins has progressed significantly. The total number of publicly available 3D structures of proteins is over 169,000 in 2022 [166]. This has laid the foundation for SBDD, where the 3D structural information is used as a guide to identify either an individual chemical or a large set of structures (including natural product structures). The very first structure-based molecular docking program was published by Kuntz [167] 40 years ago, which used the 3D structure of the target protein to guide the automated search for organic chemical structures based on geometric (and later chemical) complementarity between the molecule and the target binding site. A picture is generated here (Figure 8) to demonstrate the ligand-receptor complementarity principle for docking based on the PDB data of estrogen receptor (PDB code: 3OS8). Since then, a variety of computational docking tools have been developed. For example, MOE (Chemical Computing Group, Montreal, QC, Canada) has a docking tool, allowing the selection of placement methods and several scoring functions. Glide, another widely employed structure-based docking program, has been developed (Schrödinger, LLC, New York, NY, USA) and favorably adopted by many industry and academic users.

The docking of natural products to putative protein targets for mechanism research has been discussed in a recent book chapter [168], where Temml and Schuster presented the requirements for a successful docking study of natural products, and highlighted state-of-the-art docking studies on natural products. This work has highlighted the potential application of molecular docking to assist in the elucidation of the molecular mechanisms of natural products. This very strategy has been adopted by K.-H. Lee’s laboratory to study some tubulin inhibitors [169]. The new analogs partially inhibited colchicine’s binding to tubulin, suggesting that their binding mode could be different from that of colchicine. This hypothesis was supported by a computational docking study, which revealed a potential novel binding mode of these compounds. In another study of a set of antitumor agents [170], molecular docking of one of the compounds revealed potential binding between the compound and the active site of p38α-MAPK, indicating MAPK as a potential molecular target for the compound. Lee’s laboratory has also conducted other studies of molecular mechanisms using docking tools [60].

### 3.2. Ligand-Based Drug Discovery (LBDD)

Ligand-based drug design is an approach used in the absence of the receptor’s 3D information. It depends on the availability of compound structure–activity relationship (SAR) data. The method, known as the quantitative structure–activity relationship (QSAR) method, aims to establish a mathematical relationship between the structural descriptors of drug molecules and the measured bioactivity. The discipline of QSAR was initiated by the pioneering work of Hansch in the 1960s [171]. Since then, computing and statistical approaches have advanced significantly, although the basic concept remains the same. QSAR modeling procedures can be found in most molecular modeling software tools, such as MOE. Machine learning tools (e.g., SciKit Learn [172]) have been made available that can be employed to build quantitative models based on novel molecular descriptors (e.g., those generated by MOE).

QSAR modeling has been applied to natural product studies. In a recent publication [173], a group of researchers employed a combination of natural product library and QSAR-based virtual screening to prioritize compounds with potential antimalarial activity. Experimental validation against cultured chloroquine-sensitive and multidrug-resistant *P. falciparum* strains has confirmed the activity of two sesquiterpene lactone compounds identified via the virtual screening, demonstrating the successful use of a QSAR-based virtual screening strategy for drug discovery. This is especially useful when the disease targets are unknown. Other related work on natural product QSAR analysis can be found in [174]. We note that the QSAR technique has been widely employed to study antiviral and antitumor agents by K.-H. Lee’s laboratory in the past three decades [175,176,177], demonstrating QSAR’s applicability in the research of natural products and their derivatives.

### 3.3. Literature-Wide Association Studies (LWAS)

This is a relatively new development of computer-assisted drug discovery (CADD) approaches. It aims to analyze the scientific literature, such as PubMed abstracts or full articles, in order to discover hidden relationships among drugs, drug targets and associated diseases or conditions. The co-occurrences of biomedical terms often imply a hidden association among them. Even though the co-occurrence of a pair of terms does not necessary indicate a causal relationship, it does suggest some sort of association if the same terms occur together repeatedly. This basic concept has been codified as Word2Vec by AI researchers at Google performing text mining analysis. The tool has been released as open-source software and implemented in the SciKit Learn package [172] for all researchers who are interested in this tool.

The application of Word2Vec in drug discovery, in conjunction with simple similarity analysis or machine learning methods, has recently been described [162], where Ji et al. analyzed the PubMed abstracts related to various cancers. All drug names and various cancer names were analyzed in the abstracts using Word2Vec. The results of the analysis allowed a similarity comparison among the drugs and diseases. Based on the simple similarity values among the drugs and diseases, potential anti-inflammatory breast cancer (IBC) drugs have been proposed. Further literature analysis has supported their proposed compounds. Another interesting area of research that may be fruitful in uncovering natural products for a certain disease is the text mining of the historical medical literature. In a recent publication [178], researchers have found over 300 natural products used to treat chronic cough. Of these natural products, the 10 most frequently and continually used products were examined, and their potential should be further examined in contemporary pharmacology studies.

### 3.4. Network-Wide Association Studies (NWAS)

This is a new approach to CADD. It aims to utilize the ever-increasing amount of data that are being integrated as a knowledge graph (KG) to help uncover hidden relationships among biological concepts (such as drugs, diseases and targets). Since the relationships among drugs, targets and diseases form a network, we also term this approach network-wide association studies (NWAS). An example of such a large graph is ROBOKOP [163]. These data have captured the interactions or associations among drugs, diseases and targets, and they can be analyzed by artificial intelligence (AI) algorithms to construct models to predict unknown (hidden) relationships. The newly predicted links among drugs, diseases and targets can be proposed as hypotheses, which can be tested experimentally. The iterative process may eventually lead to the discovery of new drugs or the new use of existing drugs for a disease.

The application of such a technique to drug repurposing has been considered by Luo [179]. They developed a computational procedure (named DTINet) to predict novel drug–target interactions. This focuses on learning a low-dimensional vector representation, which accurately explains the topological properties of individual nodes in the network, and then makes a prediction based on these representations via a vector space projection scheme. Through experiments, they have validated the new interactions between three drugs and cyclooxygenase. In another publication, researchers have developed an algorithm to learn drug–disease–target relationships from knowledge graphs to propose drug repurposing hypotheses [180]. Several proposed hypotheses have been supported by literature investigations and the analysis of their potential mechanisms.

The knowledge graph approach is becoming one of the most critical tools for drug discovery and drug repurposing. For example, a knowledge graph has been built for COVID-19 research [181]. This graph has captured concurrent information about drugs, targets and COVID-19. It has laid a foundation for further NWAS-type analyses that could further support the discovery of new drugs for COVID-19. Natural product interactions in knowledge graphs are being investigated, and we expect that these knowledge graphs, combined with the NWAS methodology, would afford new opportunities for the discovery of novel uses of known natural products.

## 4. Discussion

Due to the complexities of bioactive compounds isolated from natural resources, especially those molecules with multiple chiral centers, which will have an impact on their later manufacture for market supply; the difficulties in the access to and supply of the crude materials for commercial use; concerns regarding conflicts of interest; research-related technical barriers to screening, isolation, characterization and optimization, etc., natural product drug discovery has slowed down in recent years. No new antibiotic classes have been discovered since daptomycin and linezolid in the 1980s [182], even though antibiotic resistance has been a major concern in clinics. In addition, more than 95% of the lead compounds or drugs that kill either cancer cells in culture or regress the tumors in animals have failed in Phase I clinical trials in humans [183]. Besides the high prices of chemotherapies, most drugs approved by the FDA or recommended by WHO for clinical use have little impact on the overall survival of cancer patients. The inherent slowness of working with natural products, especially regarding research on a new species that has not been studied before [184], and the challenges in the isolation of ultra-trace amounts of active components from a very small quantity of crude extract (e.g., 0.5–1 g for each species [185]) which contains millions of compounds, many pharmaceutical companies have stopped or decreased their research on drug lead isolation from natural products.

However, the rapid emergence of drug resistance has led to the re-evaluation of nature as a source to discover novel chemical skeletons to solve this problem. Strategies such as improved analytical tools, genome mining or CRISPR-Cas9, biosynthetic gene cluster identification, new engineering techniques and the rebirth of microbial culturing advances have opened up new opportunities for drug discovery from natural resources [182,186]. By using data mining, an approach using bioinformatics to identify and prioritize potential disease targets [187], or phenotypic screening, a disease-relevant target can be determined. Combinatorial chemistry approaches based on natural product scaffolds to create screening compound libraries that resemble drug-like compounds to target biological molecules can achieve a higher probability of success in lead selection for future drug development.

New technology-assisted [188,189] investigations of wider species/resources, including those that live in extreme environments, are needed to speed up the research in natural product chemistry. The prevalence of multidrug-resistant microbial pathogens (one of the main obstacles in treatment) has directed scientists to look for specialized metabolites with therapeutic properties from extreme biomes such as the gifted actinobacteria, cyanobacteria and fungi in deserts, permafrost soils and deep-sea sediments [190]. There has been evidence over the past decade that microorganisms that have adapted to life in extreme habitats are a rich source of new, specialized metabolites. It is believed that new natural products will certainly re-emerge as key starting points in drug discovery against multidrug-resistant microbials and cancer cells. In addition, to reduce the costs and time involved in the drug discovery process, the repositioning of existing naturally derived drugs to treat different diseases has gained significant attention recently [191].

## 5. Conclusions

In conclusion, recent representative lead/drug discoveries of small-molecule natural-product-based drugs against cancer and infectious diseases have been reviewed in this paper. The work in natural product chemistry is very important, as certain species of organisms are disappearing due to global environmental changes. It is urgently necessary to discover new, potent bioactive molecules from nature for future drug development, to overcome the rising drug resistance in all disease areas. The application of new research/analysis tools and advanced screening techniques may speed up the natural product lead discovery process. Mechanism studies of druggable molecules help to narrow down molecular targets for further lead improvement and drug development. 

This report also conducted a brief review of the CADD technologies, including both traditional structure-based drug design (SBDD) and ligand-based drug design (LBDD) tools, as well as emerging technologies for drug discovery, namely the text mining method of literature-wide association study (LWAS) and the knowledge graph-based network-wide association study (NWAS) techniques. The application of these emergent technologies to natural product drug discovery is still in its infancy, but the potential of these technologies could be great if one can carefully collect and curate knowledge of natural products from the medical/medicinal literature. We expect to see more such applications in the near future.

## 6. Future Directions

Molecular/genetic target-based lead optimization via chemical approaches to improve potency, decrease toxicity and overcome drug resistance is one of the main routes for future drug development. Analysis of drug candidates’ absorption, distribution, metabolism and elimination/excretion (ADME) properties will provide a direction regarding how a drug molecule could be further chemically modified, and medicinal chemists are adept in this area. It is important to take the advantage of the available data pool and technological evolution. Artificial intelligence, machine learning and plant metabolite databases [192] and compound libraries or clinical trial data analysis have been used to process large quantities of available information, which can effectively offer new research directions for existing drugs. By applying molecular biology techniques, novel bioactive compounds can be conveniently produced in bacteria or yeasts to obtain larger quantities. The further application of genomics and metabolomics to research projects may effectively accelerate the microbial natural product drug discovery process [193]. Future reports of new or reinvestigated drugs/leads should mainly include high-quality chemical structures (solubility, stability and cell-membrane permeability embedded); biochemical/biological data such as cellular/molecular targets, spectrum and safety/toxicity; potency-related data such as the minimum inhibitory concentration (MIC), minimum bactericidal concentration (MBC), half inhibitory concentration (IC_50_), half effective concentration (EC_50_), therapeutic index (TI), concentration required to reduce the viability of mock-infected cell cultures by 50% (CC_50_), selectivity index (SI, the ratio of CC_50_/EC_50_), lethal dose (LD_50_), etc., and resistance information to reduce the efforts required in resource-limited natural product research. Further, more attention should be paid to lead discovery from natural resources for the treatment of a number of neglected and rare diseases.

## Figures and Tables

**Table 1:** Selected bioactive natural products reported recently.

Compound	Structure Type	Ref #	New Bioactivity	Discovery Year	Source
8-Hydroxytubulosine	Alkaloid	[33]	Antitumor	2018	*Alangium longiflorum*
3α-Acetonyl-tabersonine	Alkaloid	[34]	Anti-drug-resistant (DR) tumor	2019	*Bousigonia mekongensis*
14,15-α-Epoxy-11-methoxytabersonine	Alkaloid	[34]	Anti-DR tumor	2019	*Bousigonia mekongensis*
Lochnerinine	Alkaloid	[34]	Anti-DR tumor	2019	*Bousigonia mekongensis*
19-I-Acetoxy-11-hydroxytabersonine	Alkaloid	[34]	Anti-DR tumor	2019	*Bousigonia mekongensis*
(−)-Neocaryachine	Alkaloid	[35]	Anti-DR tumor	2017	*Cryptocarya laevigata*
Lipojesaconitine	Diterpene/ alkaloid	[36]	Anti-tumor	2020	*Aconitum japonicum*
Taburnaemine A	Alkaloid	[37]	Anti-DR tumor	2018	*Tabernaemontana corymbosa*
Isochaihulactone	Lignan	[38]	Anti-DR tumor	2017	*Bupleurum scorzonerifolium*
Ochrocephalamines B-D	Alkaloid	[39]	Anti-HBV	2019	*Oxytropis ochrocephala*
Daphneodorins A & B	Diterpene	[40]	Anti-HIV	2020	*Daphne odora*
Kleinhospitine E	Triterpene	[41]	Antitumor/HIV	2018	*Kleinhovia hospital*
Beesioside	Triterpene	[42]	Anti-HIV	2019	*Souliea vaginata*
Myricetin	Flavonoid	[43]	Anti-SARS-CoV-2	2021	Foods
Chebulagic acid	Polyphenol	[44]	Anti-SARS-CoV-2	2021	*Terminalia chebula*
Punicalagin	Polyphenol	[44]	Anti-SARS-CoV-2	2021	Pomegranates
Ubonodin	Lasso peptide	[45]	Anti-Gram-negative bacteria	2020	*Burkholderia Ubonensis*
Bifucatriol	Diterpene	[46]	Antimalarial	2017	*Bifurcaria bifurcate*
Kakeromamide	Cyclic peptide	[47]	Antimalarial	2020	*Moorea producens*
Halymeniaol	Sterol derivative	[48]	Antimalarial	2017	*Halymenia floresii*
5,7,3′,4′-Tetrahydroxy-2′-(3,3-dimethylallyl)isoflavone	Isoflavone	[49]	Anti-SARS-CoV-2	2020	*Psorothamnus arborescens*
Gallinamide A	Depsipeptide	[50]	Anti-SARS-CoV-2	2020	Cyanobacteria *Schizothrix* and *Symploca*
Santacruzamate A	Amide	[51]	Histone deacetylase inhibitor	2013	Cyanobacteria *Symploca*
Honaucin A	Lactone	[52]	Anti-inflammatory	2012	*Leptolyngbya crossbyana*

## Data Availability

Not applicable.
